# Continuous Subcutaneous Apomorphine Infusion in Advanced Parkinson’s Disease: A Systematic Review

**DOI:** 10.7759/cureus.17949

**Published:** 2021-09-13

**Authors:** Suman Gaire, Sunam Kafle, Sanjiv Bastakoti, Anuj Paudel, Kumar Karki

**Affiliations:** 1 Department of Emergency Medicine, Palpa Hospital, Palpa, NPL; 2 Internal Medicine, College of Medical Sciences, Bharatpur, NPL; 3 Internal Medicine, Metrocity Hospital and Research Center, Pokhara, NPL; 4 Emergency Medicine, Metrocity Hospital and Research Center, Pokhara, NPL; 5 Internal Medicine, National Medical College, Birgunj, NPL

**Keywords:** : parkinson’s disease, continuous subcutaneous apomorphine infusion, device assisted therapy, advanced parkinson's, motor outcomes

## Abstract

Parkinson's disease (PD), a neurodegenerative disorder, is caused due to the loss of dopaminergic neurons in substantia nigra pars compacta, and it mainly affects the motor function of the diseased individual. The most effective treatment for PD to date is levodopa, the precursor molecule for dopamine which ultimately helps overcome the loss of dopamine in the brain. However, long-term levodopa therapy significantly impairs patients' quality of life by causing various disabling motor and non-motor complications. We conducted this study intending to review the available literature that has compared the efficacy and safety of continuous subcutaneous apomorphine infusion (CSAI) with other available treatment options like deep brain stimulation, intestinal levodopa gel, and oral dopaminergic agents. We searched PubMed, Embase, and Scopus databases using the appropriate search strategy. The studies which compared the safety and efficacy of continuous subcutaneous apomorphine infusion to other available treatment options in advanced Parkinson’s disease were included in our study. The bias assessment of the studies was done using Cochrane Risk of Bias 2.0 tool for randomized controlled trials, Risk of Bias In Non-Randomized Studies - of Interventions (ROBINS-I) tool for non-randomized interventional studies, and Joanna Briggs Institute Critical Appraisal tools (JBI) for cohort studies. We included eight articles in our systematic review including a randomized controlled trial. None of the included studies had a high risk of bias. We found that in patients with advanced Parkinson’s, CSAI demonstrated definite improvement in off-time duration. CSAI has also been shown to improve various non-motor functions, including neuropsychiatric problems in these patients. CSAI has demonstrated safety and efficacy in patients with advanced Parkinson’s disease. However, the decision-making is multifactorial. Hence, further studies are required that directly compare the available treatment options with one another and study their overall effects on patients’ quality of life.

## Introduction and background

Parkinson's disease (PD) is a progressive neurodegenerative movement disorder caused by the loss of dopaminergic neurons from the substantia nigra pars compacta, which is located in the midbrain. It is characterized by the presence of Lewy body, an eosinophilic cytoplasmic inclusion composed of aggregates of alpha-synuclein [1,2]. PD is characterized by resting tremor, bradykinesia or akinesia, rigidity, and postural instability [3]. Apart from these symptoms, the other clinical manifestations of Parkinson's disease are motor symptoms such as hypomimia, dysarthria, dysphagia, sialorrhoea, micrographia, shuffling gait, and non-motor symptoms such as autonomic dysfunction, and cognitive and neurobehavioral abnormalities. It affects 2-3% of the population older than 65 years of age, with an overall global prevalence of 0.3%, increasing with age. Globally, there are five to 35 new cases of Parkinson's disease annually per 100,000 population [4].

Patients with Parkinson's disease have more severe motor and non-motor symptoms along with the rapid and severe progression of the disease if the disease is diagnosed at an older age [5]. Diagnosis is based on clinical signs and symptoms, and various therapeutic options available for the treatment of Parkinson's disease include dopamine agonists, levodopa, anticholinergic agents, monoamine oxidase inhibitors, catechol-o-methyltransferase inhibitors, and amantadine [6]. Levodopa is the most effective treatment for Parkinson's disease combined with carbidopa to inhibit the peripheral conversion to dopamine [7]. However, long-term treatment with oral levodopa is associated with motor fluctuations and dyskinesia [8]. With a prolonged duration of treatment with levodopa, the duration of response shortens. The patients then fluctuate between the period of mobility and immobility termed as wearing off or end of dose fluctuations when they are predictable. When the fluctuations are unpredictable, and there are switches between the period of mobility and immobility, they are termed as an on-off phenomenon [9]. The disabling motor and non-motor complications in patients on long-term levodopa therapy significantly impair the patient's quality of life, and it is crucial to address the motor fluctuations and dyskinesia to improve the patient's quality of life.

Device-aided therapies available for managing patients at an advanced stage of the disease include the following: a) subthalamic deep brain stimulation (STN DBS), b) continuous subcutaneous infusion of apomorphine (CSAI), and c) continuous intestinal infusion of levodopa/carbidopa [10]. Although the exact definition of advanced Parkinson's might be debatable, advanced Parkinson's disease can be considered a poorly controlled disease despite using available first-line therapies [10]. Apomorphine is a dopamine agonist which is rapidly absorbed and has a short half-life. Apomorphine has been known to improve the off states which are not responsive to levodopa, and it helps in the dose reduction of other parkinsonian drugs [11]. It can be administered in those patients who have contraindications for deep brain stimulation and intestinal levodopa. Stibe et al. first introduced continuous subcutaneous apomorphine in Parkinson's patients who had severe on-off fluctuations [12]. The patients treated by subcutaneous infusion showed a sustained improvement in addition to the reduction in the mean duration of off periods in a day [12]. However, CSAI might be associated with various adverse effects such as nausea, cutaneous reactions, orthostatic hypotension, and autoimmune hemolytic anemia [13].

The main objective of our systematic review is to elucidate the effectiveness and adverse effects of continuous subcutaneous apomorphine infusion in patients with Parkinson's disease whose disease is poorly controlled with available first-line therapies.

Methods

We conducted the literature search, abstraction, and analysis as per Preferred Reporting Items for Systematic reviews and Meta-Analyses (PRISMA) guidelines [14].

Eligibility Criteria

We included randomized as well as non-randomized clinical trials and prospective as well as retrospective observational studies. We included studies conducted or published in any language. We excluded all studies done in pre-clinical or animal models. All review articles, commentaries, letters, editorials, book chapters, case reports, and case series were also excluded. We included patients of all ages and genders suffering from Parkinson's disease whose symptoms were not adequately controlled with oral dopaminergic agents. The intervention of interest was CSAI in patients with Parkinson's disease. Studies in which apomorphine was administered as an injection rather than continuous infusion were also excluded.CSAI was compared with placebo, oral dopaminergic agents, intestinal levodopa-carbidopa gel, or subthalamic deep brain stimulation. Studies without a comparator arm were excluded. Apomorphine injection was not considered a comparator for our study. The primary outcomes of interest were motor outcomes. The motor outcomes were assessed with Unified Parkinson's Disease Rating Scale (UPDRS) score, off-time duration, and Abnormal Involuntary Movement Scale (AIMS) score. The studies which did not include any of the motor outcomes of interest were excluded. The secondary outcomes of our study were other non-motor outcomes like Mini-mental state examination (MMSE) score, Non-Motor Symptoms Scale (NMSS), Neuropsychiatric Inventory (NPI) score, Hamilton Depression rating scale-17 (HAMD-17), and adverse effects of the treatment.

Information Sources

Two independent reviewers searched for articles on multiple databases, including PubMed, Scopus, and Embase, from inception to 2021 July 15. All databases were searched on 2021 July 15, and the retrieved articles were imported to Covidence software for screening.

Search Strategy

The key search terms "Parkinson's disease", "Parkinsonism", "Advanced parkinsonism", and "Apomorphine infusion" were used in combination with BOOLEAN operators "OR" and "AND" to search for relevant articles. We applied the filter 'humans' while searching on PubMed. No other filters or automated tools were used during the literature search.

Selection Process

**Figure 1:**
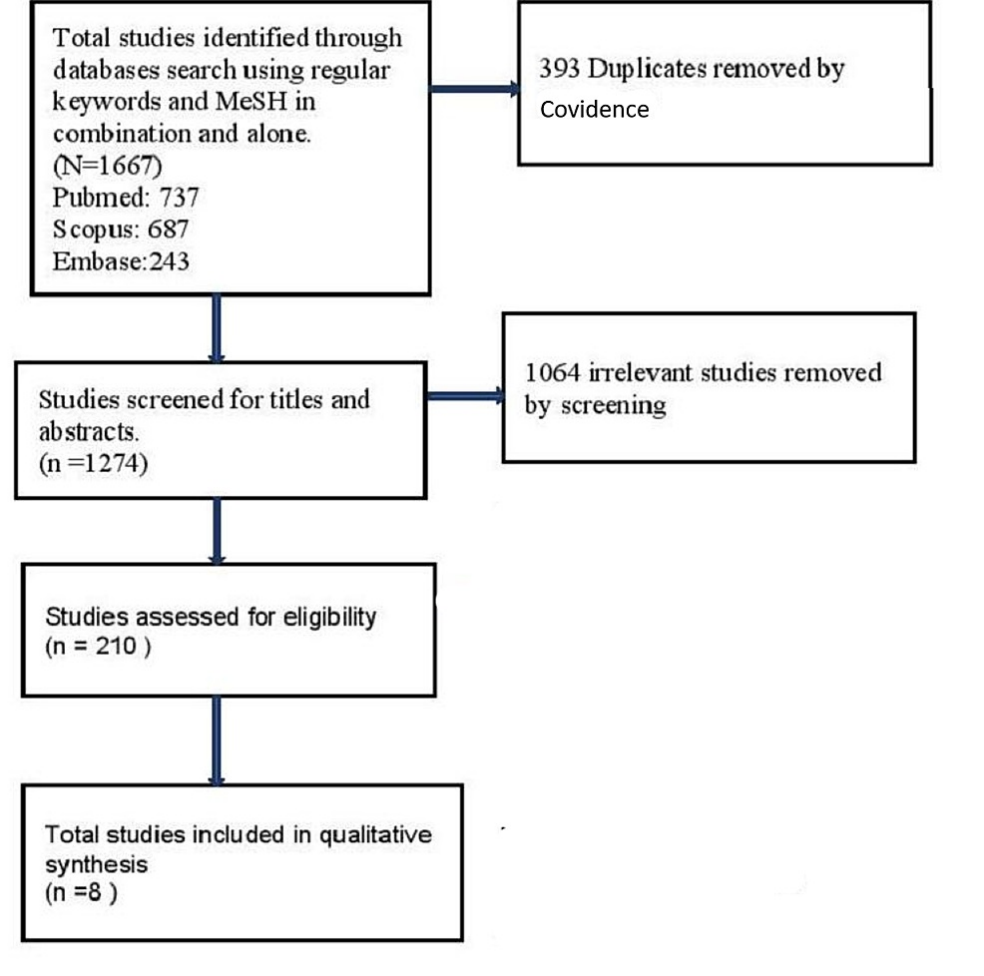
Preferred Reporting Items for Systematic reviews and Meta-Analyses (PRISMA) flow diagram of our selection process

The initial literature search on PubMed, Scopus, and Embase identified a total of 1667 articles. A total of 393 articles were identified as duplicates by Covidence and were removed automatically. Titles and abstracts of 1274 articles were reviewed by two reviewers independently, and they excluded 1064 irrelevant articles. Two reviewers again screened the full text of the remaining 210 relevant articles to check if the studies fit our eligibility criteria. We removed 202 articles for various reasons, as mentioned in the Prisma flow diagram (Figure 1). A total of eight studies that fulfilled our eligibility criteria were included in our review. Conflicts at both stages of screening were resolved by the consensus of the panel of all five reviewers.

Data Collection Process

One reviewer collected the data, and another reviewer cross-checked it. Relevant data from the included studies, including the study characteristics and outcome parameters, were collected in a separate excel sheet. All five reviewers contributed to data collection, and they worked independently. No automated tools were used for the data collection process.

Study Risk of Bias Assessment

To assess the risk of bias of the included studies, we used the Risk of Bias 2 (RoB 2) tool for randomized controlled trials [15], Risk of Bias In Non-Randomized Studies - of Interventions (ROBINS-I) tool for non-randomized interventional studies [16] and Joanna Briggs Institute Critical Appraisal tools (JBI) for cohort studies [17]. No studies were excluded based on bias assessment.

## Review

Results

We have included eight studies in our systematic review after careful screening and exclusion of studies that did not meet our eligibility criteria. Five of these studies are prospective cohort studies [18-22]. Studies by Di Rosa et al. and Morgante et al. are the two reports of the same open-labeled parallel-group trial published after a year and two years of initiation of the trial, respectively [23,24]. One of the studies is a double-blinded randomized controlled trial [25]. The study population of six of the studies had Parkinson's disease for a minimum of 10 years [18,21,22,24,25]. The exact duration of the illness was not mentioned in the studies by Antonini et al. and Martinez-Martin et al. [19,20]. In all the included studies, apomorphine was given subcutaneously in continuous infusion form. The only randomized controlled trial (RCT) included in our study had compared the effect of apomorphine to placebo, while all other studies had various treatment modalities as comparators [25]. The duration of the studies varied from 12 weeks to five years. The characteristics of included studies, including the dose of apomorphine and the comparator, are mentioned in Table 1.

**Table 1:** Characteristics of included studies CSAI: continuous subcutaneous apomorphine infusion, IJLI: Intra Jejunal levodopa infusion, STN-DBS: Subthalamic Nucleus deep brain stimulation, T= Treatment, C=Comparator, RCT: Randomized controlled trial, SD: standard deviation, NA: not available

SN	Study	Study design	Disease duration mean±SD	Intervention (dose of CSAI)	Comparator	Duration of study
1.	Morgante et al. 2004/ Di Rosa et al. 2003 [23,24]	open-labeled, parallel group trial	T=20 ± 36 months C =122 ± 3 months	100mg/day over 6-8 mg/hour (mean ± SD)	Oral dopaminergic drugs	2 years/1 year
2.	De Gaspari et al. 2006 [18]	prospective cohort study	T= 10 ± 5 years C= 12 ± 2.45 years	78 ± 24.42 mg/day (mean± SD).	STN-DBS, performed using stereotactic surgery	12 months
3.	Antonini et al. 2010 [19]	prospective cohort study	NA	83.4 ± 19.2 mg over 14 ± 2 Range 70-112.5 mg per day over 10–16 hrs (mean± SD.)	STN-DBS, performed using stereotactic surgery	Five years
4.	Martinez-Martin et al. 2011 [20]	prospective cohort study	NA	12-16 hrs Per day Dose: NA.	Best Conventional therapy for the patients	NA
5.	Martinez-Martin et al. 2015 [21]	prospective cohort study	T= 14 ± 4.4 years C= 16.1 ± 6.7 years	105.9 ± 23.2 mg/day over 15.9 ± 3.5 hrs/day (mean ± SD)	IJLI: 1,815.4 ± 771.5 mg/day for 17.3 ± 3.6 hrs/day	NA
6.	Katzenschlager et al. 2019 [25]	Double blinded RCT	T= 11·8 ± 5·6 years C= 10.6 ± 4·3 years	3–8 mg/hrs over 16 hrs a day (range 14–18 hrs)	placebo saline infusion, 16 h a day, (range 14–18 hrs)	12 weeks
7.	Dafsari et al. 2019 [22]	Prospective cohort study	T=13.5 ± 5.6years C1= 10.7 ± 4.8 years C2=14.6± 5.3 years	15.4 ± 2.6 hours/day (mean± SD)	C1: STN-DBS C2: IJLI for 15.4 ± 1.3 hours/day	6 months

The total number of study participants of all the studies included in our review is 477, out of which 187 people received CSAI, and 290 people received alternative treatment or placebo (Table 2). The overall mean and standard deviation (SD) of the people receiving CSAI is 61.3±10.5 years, and that in the comparator group is 62.3±8.9 years (Table 1). The demographic features of the included studies are demonstrated in Table 2.

**Table 2:** Demographic characteristics of the included studies

SN	Author	Year of Publication	Total study population (N)	Participants in Treatment group (T)	Participants in comparator group (C)	Age of T arm (Mean ± SD), in years	Age of C arm (Mean ± SD), in years
1.	Morgante et al./ Di Rosa et al. [23,24]	2004/2003	27	10	17	54 ± 9	56 ± 8
2.	De Gaspari et al. [18]	2006	25	13	12	59 ± 13	60.5 ± 6.5
3.	Antonini et al. [19]	2010	25	12	13	58 ± 12	61 ± 8
4.	Martinez-Martin et al. [20]	2011	34	17	17	59.5 ± 11.7	66.4 ± 7.0
5.	Martinez-Martin et al. [21]	2015	87	43	44	62.3 ± 10.6	62.7 ± 9.1
6.	Katzenschlager et al. [25]	2019	106	53	53	63.6 ± 9.3	63 ± 8.3
7.	Dafsari et al. [22]	2019	173	39	101 and 33	61.6 ± 9.8	61.5 ± 9.5 and 65.4 ± 8.8
Total			N=477	T=187	C=290	61.3 ± 10.5 years	62.3 ± 8.9 years

Motor Outcomes

The motor effects of continuous apomorphine infusion were our primary outcome of interest. Hence, all the included studies have reported motor outcomes. However, only a single randomized control trial reporting motor outcomes was identified. In the RCT included, Katzenschlager et al. 2018, the study's primary endpoint was an absolute change in off-time duration throughout follow-up duration of 12 weeks. The study reported a significant decrease in off-time hours per day in the apomorphine infusion arm compared to the placebo arm, the difference being -1·89 (-3·16 to -0·62) hours with a 95% confidence interval. Furthermore, 62% of patients in the apomorphine arm experienced more than two hours of reduction of off time from baseline compared to 29% for placebo. Also, the on-time troublesome dyskinesia free period was higher in the apomorphine group (2·77 ± 3·26 hours) as compared to the placebo group (0·80 ± 2·93 hours). However, no significant difference was found in both groups on UPDRS III motor scores during the on period [25].

Apart from Morgante et al., 2004 and Di Rosa et al., 2003 [23,24], which have only reported AIMS scoring, all other included studies have reported UPDRS III in their outcomes. Three studies have reported UPDRS IV as well [20-22]. The motor outcomes of the included studies are summarized in Table 3.

**Table 3:** Motor outcomes of included studies CSAI: continuous subcutaneous apomorphine infusion, IJLI: Intra Jejunal levodopa infusion, STN-DBS: Subthalamic Nucleus deep brain stimulation, UPDRS: Unified Parkinson's Disease Rating Scale,  AIMS: Abnormal Involuntary Movement Scale, P: p-value

Study		Di Rosa et al. 2003 [23]	Morgante et al. 2004 [24]	De Gaspari et al. 2006 [18]	Antonini A. et al. 2010 [19]	Martinez-Martin et al. 2011 [20]	Martinez-Martin et al. 2015 [21]	Katzenschlager et al. 2018 [25]	Dafsari et al. 2019 [22]
Off time (CSAI)	Baseline	awake duration: 5 ± 1.52	awake duration: 5.0 ± 1.6	reduction (h/day): 2.8 ± 0.8	mean reduced by 49%			Change (h/day) : –2·47 ± 3·70	
	Follow up	1-year: 2 ± 0.4 (P < 0.01)	2-year 2.0 ± 0.5 (P < 0.01)	1-year 1.4 ± 0.5 (P < 0.001)					
Off time (Comparator)	Baseline	L-dopa: 6 ± 1.70	L-dopa 6.5 ± 1.8	STN-DBS 3.1 ± 1	STN-DBS: mean reduced by 91%			Placebo: –0·58 ± 2·80 (P = 0·0025)	
	Follow up	1-year: 6.5 ± 1.51	2-year 6.7 ± 1.8	1-year: 0.8 ± 0.7 (P < 0.001)					
UPDRS III (CSAI)	Baseline			‘’off’’ Score: 32.1 ± 7.3	"on" score: 24.2 ± 10	36.94 ± 11.42	30.79 ± 10.40	Change during on periods: –3·42 ± 11·69	29.5 ± 11.0
	Follow up			1-year: 32.9 ± 8.5	1 year: 21.1 ± 8.6; Last follow up: 20.9 ± 14.	5.35 ± 8.21 (P = 0.0003)	6-months: 17.46 ± 8.08 (P < 0.0001)		6-months: 27.8 ± 10.1
UPDRS III (Comparator)	Baseline			33.5±12.9	19.4 ± 7.6	20.06 ± 9.68	IJLI: 27.29 ± 12.28	Change during on periods Placebo: –0·89 ± 9·73 (P = 0·4642)	STN DBS: 23.9 ± 11.4; IJLI: 29.8 ± 12.3
	Follow up			1-year: 15.7 ± 7 (P < 0.003)	1-year: 18.7 ± 9.6; Last follow up: 20.2 ± 8.3	19.35 ± 12.80 (P = 0.69)	6-months: 15.07 ± 10.37 (P < 0.0001)		STN DBS: 6-month: 23.0 ± 11.0; IJLI 6 months: 27.8 ± 11.0
UPDRS IV (CSAI)	Baseline					10.00 ± 6.43	Baseline: 10.02 ± 4.68; 6-months: 5.93 ± 3.35 (P < 0.0001)		9.0±4.7
	Follow up					3.53 ± 3.52 (P = 0.0003)			6-month 5.9 ± 3.6 (P < 0.001)
UPDRS IV (Comparator)	Baseline					7.93 ± 5.43	9.93 ± 3.29		STN DBS: 6.3 ±3.7; IJLI : 9.6 ± 3.5;
	Follow up					7.00 ± 4.46 (P = 0.48)	6-months: 4.36 ± 3.07 (P < 0.0001)		STN DBS: 6-months: 5.9 ± 3.6 (P < 0.001) IJLI 6-months: 5.3 ± 2.6 (P < 0.001)
Other outcomes (CSAI)	Baseline	AIMS: 7.7 ± 1.2	AIMS : 7.7 ± 1.2	AIMS: 9.1 ± 2.8	no significant difference in dyskinesia duration			Change in On-time without troublesome dyskinesia (h per day): 2·77 ± 3·26	
	Follow up	1-year: 4 ± 0.6 (P < 0.01)	2-year 4.0 ± 0.6 (P < 0.01)	1-year: 9.4 ± 3.1					
Other outcomes (Comparator)	Baseline	7.9 ± 1.3	7.7 ± 1.4	10.2 ± 2.9	(5-year grand mean) 80% reduction of dyskinesia duration and 83% reduction of dyskinesia disability			Placebo: 0·80 ± 2·93 (P = 0·0008)	
	Follow up	1-year: 8 ± 1.3	2-year 7.9 ± 1.6	1-year 1.9 ± 1.1 (P < 0.001)					

Non-motor and Other Outcomes

All of the included studies have studied the effect of continuous apomorphine in non-motor symptoms. The studied non-motor symptoms include Mini-Mental State Examination (MMSE) score, Non-Motor Symptoms Scale (NMSS) Neuropsychiatric Inventory (NPI) score, Hamilton Depression rating scale-17 (HAMD-17), PHQ-8 score, Brief Psychiatric Rating Scale (BPRS), Patient Global Impression of Change (PGIC), Levodopa Equivalent Dose (LEDD) and Beck Depression Index (BDI).

An RCT by Katzenschlager et al. in 2018 showed a significant improvement in PGIC scores and reduction of levodopa equivalent dose in APO treated patients vs. placebo (p<0.05). However, the study didn't show a significant change in PHQ-8 score in APO treated patients vs. placebo (p>0.05) [25]. The results from Martinez-Martin et al. in 2015, Martinez-Martin et al. in 2011, and Dafsari et al. in 2019 showed significant improvement in NMSS (calculated in various domains) in APO-treated patients [20-22]. The studies showed significant improvement in PHQ-8 score, LEDD, and BDI in APO-treated patients. The non-motor outcomes are summarized here in Table 4.

**Table 4:** Non-motor outcomes of included studies CSAI: continuous subcutaneous apomorphine infusion, IJLI: Intra Jejunal levodopa infusion, STN-DBS: Subthalamic Nucleus deep brain stimulation, Mini-mental state examination (MMSE) score, Non-Motor Symptoms Scale (NMSS) Neuropsychiatric Inventory (NPI) score, Hamilton Depression rating scale-17 (HAMD-17), PHQ-8 score, Brief Psychiatric Rating Scale (BPRS), Patient Global Impression of Change (PGIC), Levodopa Equivalent Dose (LEDD)  Beck Depression Index (BDI), P: p-value

Study		D De Gaspari et al. 2006 [18]	Antonini A. et al. 2010 [19]	Martinez-Martin et al. 2015 [21]	Martinez-Martin et al. 2011 [20]	Morgante et al. 2004 [24]	Katzenschlager R et al. 2018 [25]	Dafsari et al. 2019 [22]	Di Rosa et al. 2003 [23]
Equivalent dose of levodopa	CSAI	Baseline: 665.98 ± 215 mg/day					Change (mg): –492·1 ± 618·3 (P = 0·0014, significant)		
		Follow-up: 470 ± 229 mg/day (−29%, P < 0.034)							
	Comparator	STN DBS Baseline: 980 ± 835						–163·7 ± 367·5	
		Follow-up: 374 ± 284 mg/day (−62%, P < 0.003).							
PDQ 8	CSAI					Baseline: 49.85 ± 16.59	Baseline: 55.70 ± 19.80	Change: –0·06 ± 14·37 (P = 0·3971, not significant)	Baseline: 43.5 ± 19.4
						Follow-up: 35.03 ± 18.00 (P < 0.0001)	Follow up: 32.35 ± 21.54 (P=0.001)		Follow up: 30.3 ± 17.0 (P < 0.001)
	Comparator					IJLI Baseline: 48.58 ± 14.62	Conventional Baseline: 35.84 ± 23.10	Placebo: 2·40 ± 11·83	STN-DBS Baseline: 37.6 ± 16.4; Follow up: 27.5 ± 15.6 (P < 0.001)
						Follow-up: 31.96 ± 14.89 (P < 0.0001)	Follow up: 44.85 ± 17.57 (P = 0.02)		IJLI Baseline: 47.7 ± 18.2; Follow up: 37.6 ± 13.9 (P < 0.001)
NMSS	CSAI					Baseline: 82.37 ± 49.54	Baseline: 105.94 ± 65.43:		Baseline: 76.3 ± 54.2
						Follow-up: 56.21 ± 32.21(P = 0.0007)	Follow up: 56.94 ± 45.39 (P = 0.0003)		Follow up: 54.2 ± 36.8 (P = 0.009)
	Comparator					Baseline: 90.95 ± 45.00	Baseline: 47.65 ± 43.40;		STN DBS: Baseline: 56.2 ± 32.8; Follow up: 38.9 ± 23.6 (P < 0.01)
						Follow-up: 53.66 ± 38.67 (P < 0.0001)	Follow up: 52.00 ± 37.65 (P = 0.22)		IJLI: Baseline: 86.9 ± 45.5; Follow up: 62.1 ± 37 (P = 0.02)
MMSE	CSAI		Baseline: 29 ± 2				Baseline: 27.6 ± 2.2;		
			one year: 28 ± 2; last f/u: 29 ± 2 (P > 0.05)				Endpoint: 27.4 ± 2.1 (not significant)		
	Comparator		STN DBS Baseline: 29 ± 2				Oral dopaminergic drugs: Baseline: 27.5 ± 2.0		
			one year: 29 ± 1; last f/u 29 ± 1 (P > 0.05)				Endpoint: 27.2 ± 2.0 (not significant		
NPI	CSAI	No significant difference was seen.	Baseline: 10 ± 15						
			one year: 10 ± 11; last f/u: 12 ± 11 (P > 0.05)						
	Comparator	Baseline: 6.58 ± 9.8	Baseline: 6 ± 9						Baseline: 27 ± 7.6
		Follow-up: 18.16 ± 10.2 (P < 0.02)	one year: 13 ± 18; last f/u: 13 ± 12 (P < 0.05)						Endpoint: 25 ± 7.8 (P > 0.05)
BPRS	CSAI						Baseline: 28.0 ± 7.4		Oral dopaminergic Baseline 26 ± 7.4
							Endpoint: 26.5 ± 7.3 (not significant)		Endpoint: 25 ± 7.4 (P > 0.05)
	Comparator						Baseline: 26 ± 7.6;		
							Endpoint 3 26 ± 7.1 (not significant)		
Other outcomes	CSAI	CF: No significant difference seen.	HAMD-17: Baseline: 10 ± 7		Sexual functioning Baseline: 2.56 ± 5.29		BDI Baseline: 22.0 ± 6.0	PGIC: 3·23 ± 1·42 (P < 0·0001, significant)	BDI: Baseline 21 ± 6.2
			one year: 7 ± 6; last f/u: 7 ± 9 (P > 0.05)		Follow-up: 1.93 ± 3.59 (P < 0.18)		End-point: 10.0 ± 2.6 (P < 0.001)		End-point 10 ± 2.6 (P < 0.001)
	Comparator	Baseline: 43.58 ± 7.83;	Baseline: 5 ± 3		Baseline: 5.73 ± 7.93		Baseline: 20 ± 2.7	4·43 ± 1·10	Baseline: 19 ± 2.8
		Follow-up: 36.58 ± 10.23	one year: 8 ± 7; last f/u: 8 ± 4 (P > 0.05)		Follow-up: 2.32 ± 4.12 (P = 0.014)		Endpoint: 21 ± 2.8 (not significant)		End-point: 20 ± 2.8 (P > 0.05)

Adverse Effects

Nausea and local site discomfort or subcutaneous nodules are the most common side effects observed among patients receiving apomorphine. Side effects like infusion site erythema (9/53), dyskinesia (8/53), headache (7/53), insomnia (6/53) were reported in the RCT by Katzenschlager et al. in the treatment arm [25]. Three of the included studies had not mentioned the side effect profile of the treatment or comparator [18-20]. Side effects of the various interventions mentioned in five of the included studies are demonstrated in Table 5.

**Table 5:** Side effects of the interventions in the included studies CSAI: continuous subcutaneous apomorphine infusion, IJLI: Intra Jejunal levodopa infusion, STN-DBS: Subthalamic Nucleus deep brain stimulation NA: not available

Author	Side effects						
	Subcutaneous nodules or local site discomfort	Nausea	Stoma site irritation	Tube dislocation	Somnolence	Events related to surgery or device	Wound healing disturbance
Morgante et al./ Di Rosa et al. [23,24]	10/10 in CSAI group, 0/17 in dopaminergic group	1/10 in CSAI group, 0/17 in oral dopaminergic group	NA.	NA	NA	NA	NA
Martinez-Martin et al. [21]	NA	3/43 in CSAI group and 0/44 in IJLI	0/43 in CSAI group and 8/44 in IJLI group	NA in CSAI group, 9/44 in IJLI group	3/43 in CSAI group, 0/43 in IJLI group	NA	NA
Katzenschlager et al. [25]	24/53 in CSAI group, 0/53 in placebo group	12/53 in CSAI group, 5/53 in placebo group	NA	NA	NA	NA	NA
Dafsari et al. [22]	NA	NA	NA	NA	NA	0/39 in CSAI group, 2/101 in STN-DBS group, 2/33 in IJLI group	0/39 in CSAI group, 3/101 in STN-DBS, 2/33 in IJIL group

Discussion

In this systematic review, we compared the efficacy and safety of continuous apomorphine infusion with oral dopaminergic agents, intestinal levodopa-carbidopa gel, and subthalamic deep brain stimulation in patients with advanced Parkinson's disease.

The efficacy of subcutaneous therapy in off periods has been well proven over time. Subcutaneous apomorphine has been used as rescue therapy for severe off periods in Parkinson's disease [26]. The beneficial effects have also been seen with continuous apomorphine infusion. Three studies have compared the effects of CSAI as compared to conventional medical treatment [23-25]. All of them have reported a decrease in off-time duration as compared to conventional medical treatment. Two studies have compared the off-time duration between CSAI and deep brain stimulation [18,19]. Both of the studies have reported greater benefit in reducing off time duration by STN DBS as compared to CSAI. Antonini et al. was a prospective study of five years duration [19]. In the study, only two out of 12 patients in the CSAI group reached the five-year follow-up for various reasons. However, in the STN DBS group, 12 out of 13 patients reached the follow-up duration of five years [19]. Therefore, the results may have to be interpreted with caution. On the other hand, De Gaspari has shown clear benefits of STN DBS over CSAI in off-time duration during 12 months of follow-up duration [18]. We could not find any studies which compared the off-time duration between CSAI and LCIG.

Movement Disorder Society-Unified Parkinson's Disease Rating Scale is a widely used tool to assess Parkinson's disease patients. MDS UPDRS has four parts; Part I: Non-Motor Aspects of Experiences of Daily Living (nM-EDL), Part II: Motor Aspects ofExperiencesofDaily Living (M-EDL), Part III: Motor Examination, and Part IV: Motor Complications [27]. The majority of studies have reported UPDRS III ( motor examination). Two studies have reported UPDRS III in CAI vs. Placebo [20,25]. Martinez Martini reported significant improvement in UPDRS III scores in the CSAI arm with no difference in the placebo arm [20]. However, this was a non-randomized study, and the baseline UPDRS III of patients in the CSAI group was significantly higher than in the placebo group. In the RCT, the difference in UPDRS III scores between CSAI and placebo arm was not seen [25]. UPDRS III did not improve during on periods on the patients treated with STN DBS [19]. However, STN DBS improved UPDRS III scores significantly during the off period, whereas CSAI did not study by De Gaspari et al. [18]. STN DBS significantly improves the off period in patients while not having many effects in the on period [28].

The TOLEDO trial has shown a significant decrease in dyskinesia duration with CSAI, whereas Antonini et al. failed to show any significant difference [19,25]. The TOLEDO trial was only 12 weeks duration whereas Antonini et al. reported outcomes up to five years [19,25]. This might have caused the difference in findings. Previous studies have suggested that the effects of CSAI on dyskinesia are transient [29,30]. The effects of STN DBS on dyskinesia seem to be significant and last longer [19,31]. AIMS score also evaluates the dyskinesia in Parkinson's disease. De Gasperi et al. have shown a substantial decrease in AIMS score with STN DBS with no change in the CSAI group [18]. UPDRS IV scores tend to decline with treatment from CSAI, IJLI, or STN DBS [20-22].

The effects of continuous apomorphine infusion on non-motor symptoms were studied in various cohort studies, open-label studies, and an RCT. Three cohort studies looked at NMSS scores, and the results were consistent with the significant improvement in the NMSS score in the APO group [20-22]. There is only a single RCT measuring the non-motor effects of APO. The results from the RCT by Katzenschlager et al. in 2018 showed significant improvements in PGIC and reduction of levodopa equivalent dose in APO treated patients vs. placebo (p<0.05). The study didn't show a significant change in the PHQ-8 score [25]. However, there was a substantial improvement in the PDQ-8 scores in three cohort studies. This suggests we need to have more studies to determine the effect of APO on PDQ-8 scores. APO infusion also has shown to reduce the equivalent levodopa dose significantly and BDI index; however, there was no significant change in BPRS, HAMD-17, and MMSE score [18,23].

Apomorphine is given on continuous infusion form; therefore, local site reactions such as discomfort, erythema, and subcutaneous nodules are the most frequently reported side effects of CSAI therapy. Nausea and vomiting experienced by the patient after receiving apomorphine were efficiently controlled by giving prophylactic domperidone before initiating the treatment [23-25]. In the study by Morgante et al., all patients receiving CSAI had developed itchy nodules at the infusion site, which was improved by the application of steroid ointment and with the dilution of the apomorphine [24]. Other side effects such as dyskinesia, headache, insomnia, and somnolence were reported by fewer participants in the RCT conducted by Katzenschlager et al. [25]. All adverse events that caused the patients to withdraw from this study were reversed with cessation of treatment, and none of them had any long-term effects [25]. CSAI was compared to IJLI in two studies where stoma site irritation, tube dislocation, abdominal bloating, peritonitis, and wound healing disturbances were reported as side effects to the participants receiving IJLI [21,22]. Patients who received STN-DBS as the intervention were reported as having side effects related to the surgery [22].

Overall, CSAI is usually well-tolerated by the patients and has shown to have a safer side effects profile compared to the other interventions, including IJLI and STN-DBS [21-25]. The most common side effects reported by the patients seem to be due to the route of administration, which could be managed with simple interventions. However, current literature does not have enough long-term studies comparing the safety profile of CSAI with other treatment options and placebo. A better understanding of any treatment choice's side effects profile is essential for physicians to practice patient-centered evidence-based medicine and for a patient to make a better-informed decision. Hence, we suggest conducting more studies with a longer duration of time to explore the side safety profile of CSAI in patients who have advanced Parkinson's disease.

This study had several strengths. It is the first systematic review that has studied the effects of continuous apomorphine infusion compared to other treatments in advanced Parkinson's disease. A thorough literature search was done. Only studies with comparator groups were included in our study, and none of the included studies had a high risk of bias.

There were several limitations of our study. We were able to collect data from only eight studies that fulfilled our inclusion criteria. The implementation of strict selection criteria limited the number of articles to only eight. There was only one double-blind randomized controlled trial where patients from the 23 different European hospitals were included. Apart from a single RCT which was included in other studies, patients were not randomized and blinded. There are no evidence-based, widely accepted guidelines regarding the dose and duration of the treatment with apomorphine infusion. Different studies had different criteria for selecting patients for apomorphine infusion. In addition, there is no uniformity in the doses and duration of treatment with apomorphine in the included studies. These factors make it difficult to compare the results of the study and derive conclusions.

The selection of treatment modality for advanced Parkinson's is a complex decision process. Various factors, including cost-effectiveness, availability of treatment options, should be considered, along with the efficacy and side effect profile of the treatment options. Shared decision-making with the patients remains crucial for the success of the treatment. However, adequate information does not seem to be available to facilitate the shared decision process to maximize a favorable outcome. Therefore, multiple studies are necessary, which help to clarify the difference in safety and efficacy of available treatment options. Furthermore, studies that directly compare the available treatment options with each other in terms of patient-centric outcomes and quality of life seem to be the need of time. 

## Conclusions

This study is the first review to compare among Parkinson's disease patients the efficacy and safety of CSAI with placebo and other conventional treatment modalities such as STN-DBS or levodopa. PD is a neurodegenerative disorder characterized by resting tremor, bradykinesia or akinesia, rigidity, and postural instability. CSAI has shown to be effective in improving motor function in off period among Parkinson's patients. With the tolerable side effects profile and significant improvement in motor and non-motor outcomes among patients with advanced Parkinson's disease, CSAI therapy could help as an adjuvant therapy to conventional treatment. However, there is only a single RCT comparing its efficacy to the placebo and not a single one comparing it to other therapies. Further clinical trials with direct comparison among available treatment options for advanced Parkinson's disease should be conducted to better understand the differences in outcomes of the treatment.
